# The Impact of Blood Lead and Its Interaction with Occupational Factors and Air Pollution on Hypertension Prevalence

**DOI:** 10.3390/toxics12120861

**Published:** 2024-11-27

**Authors:** Yajun Gong, Ying Wang, Qiying Nong, Peixia Hu, Zhiqiang Li, Xiangyuan Huang, Meimei Zhong, Xinyue Li, Shaomin Wu, Fangfang Zeng, Na Zhao, Yiru Qin, Suhui Liu, Jiaying Hong, Ligang Hu, Wangjian Zhang, Yongshun Huang

**Affiliations:** 1 Guangdong Province Hospital for Occupational Disease Prevention and Treatment, Guangzhou 510300, China; gongyajun1103@163.com (Y.G.); nongqy@mail3.sysu.edu.cn (Q.N.); hupeixia9@163.com (P.H.); lizhq78@mail2.sysu.edu.cn (Z.L.); huangxy@gdpcc.com (X.H.); zhongmm@gdpcc.com (M.Z.); lixy753@mail2.sysu.edu.cn (X.L.); zhaonabmu@126.com (N.Z.); yqin@gdoh.org (Y.Q.); liush359@mail2.sysu.edu.cn (S.L.); hjyttkx@163.com (J.H.); 2 School of Public Health, Southern Medical University, Guangzhou 510515, China; 3 Department of Medical Statistics, School of Public Health & Center for Health Information Research & Sun Yat-Sen Global Health Institute, Sun Yat-Sen University, Guangzhou 510080, China; wangy633@mail2.sysu.edu.cn (Y.W.); wushm@mail.sysu.edu.cn (S.W.); zengff@mail.sysu.edu.cn (F.Z.); 4 School of Public Health, Shanxi Medical University, Taiyuan 030001, China; 5 State Key Laboratory of Environmental Chemistry and Ecotoxicology, Research Center for Eco-Environmental Sciences, Chinese Academy of Sciences, Beijing 100085, China; lghu@rcees.ac.cn

**Keywords:** lead, hypertension, blood pressure, air pollution, occupational exposure

## Abstract

Large-scale epidemiological studies on the association of blood lead levels with blood pressure and hypertension prevalence are still limited, particularly among lead-exposed workers. The evidence is even more scarce on the interaction of blood lead levels with occupational variables and ambient air pollution levels. We developed mixed-effect models based on a large group of lead-exposed workers (N = 22,002). The results were also stratified by multiple groupings. Compared to participants with blood lead < 20 μg/L, those with levels > 20 μg/L had a 26–37% higher prevalence of hypertension, as well as a 0.65–13.7 mmHg higher systolic and diastolic blood pressure. Workers exposed to high PM10 levels had a 21–28% higher risk. Workers exposed to high temperatures had a 0.41–2.46 mmHg greater increase in blood pressure, and those not exposed to dust had a 1.29–1.65 mmHg greater blood pressure increase. Our findings suggested the negative impact of blood lead on blood pressure and the prevalence of hypertension, with workers exposed to high PM10 concentrations, those exposed to occupational high temperature, and those without dust exposure being more vulnerable.

## 1. Introduction

Due to its widespread use and environmental contamination, lead has multiple sources and pathways of exposure. Chronic lead exposure can lead to premature deaths, disease burden, and persistent negative effects on health [1,2]. According to estimates, in 2019, lead exposure resulted in a loss of 21.7 million disability-adjusted life years (DALYs) globally, including 4.6% of the burden of cardiovascular disease [3]. Among all subtypes of cardiovascular disease (CVD), hypertension and high blood pressure had the highest disease burden [4]. The World Health Organization estimated that a staggering 1.4 billion people worldwide are afflicted with hypertension [5]. An estimated 245 million adults have been diagnosed with hypertension in China alone [6]. This issue is particularly significant among workers who are exposure to higher concentrations of hazardous occupational factors. A large-scale epidemiological survey showed that, among Chinese workers, the prevalence of hypertension reached a staggering 25.7% [7], significantly surpassing the prevalence observed in the same age groups in the general population.

Previous epidemiological studies have identified lead exposure as a notable risk factor for hypertension in the general population [8,9], although the underlying biological mechanisms remain unclear. Some studies have investigated the association between lead exposure and hypertension among workers; however, the evidence is still limited and the conclusions are inconsistent. For instance, a study in Kenya reported a significant association between blood lead levels (BLLs) and changes in blood pressure [10] whereas a cohort study in the United States showed no association between hypertension and elevated blood lead from occupational sources [11]. Furthermore, existing studies may be challenged by small sample sizes and limited statistical power. Therefore, to clarify the hypertensive effect of BLLs among Chinese workers, an epidemiological study encompassing a large sample size is imperative.

In addition to lead, the workers may also be exposed to other occupational hazards, such as dust, noise, high temperature, and benzene, toluene, ethylbenzene, and xylenes (BTEX). The hypertensive impact of these hazards have been suggested in multiple previous studies [12,13,14,15]. Ambient air pollution may also play a significant hypertensive role among workers. The potential mechanisms include the notion that the indoor air quality largely depends on the ambient environment, particularly due to ventilation [16,17]. Additionally, workers engaged in outdoor activities may be directly exposed to ambient air pollution [18]. These occupational and environmental factors may interact with the blood lead to affect the risk of hypertension. However, existing studies primarily focus on the individual effects of these factors, while ignoring the interaction of blood lead with these factors on the prevalence of hypertension.

Therefore, we explored the association of BLLs with blood pressure and the prevalence of hypertension, and investigated the interaction effects of BLLs with occupational exposures and air pollutants in this study, based on the physical examination data of a large group of workers in South China.

## 2. Materials and Methods

### 2.1. Study Population

**Figure 1:**
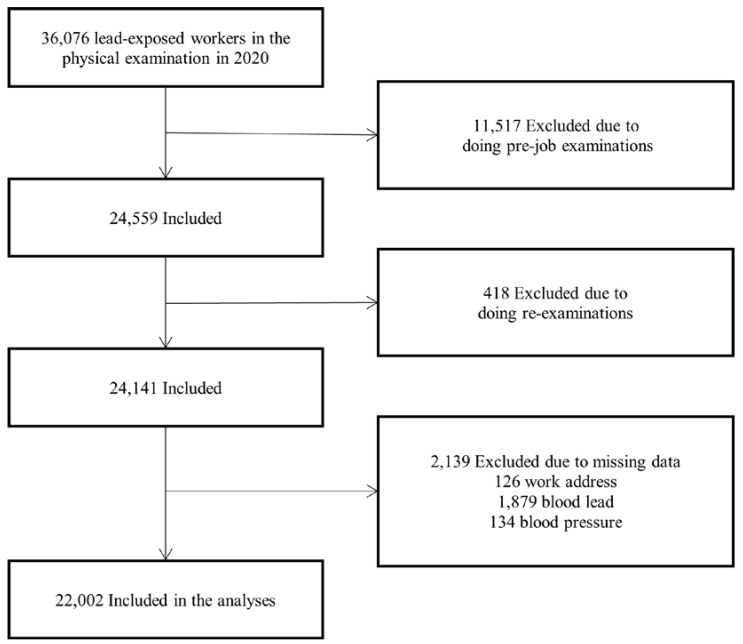
Flowchart of participant inclusion and exclusion.

The present research was conducted based on the data collected from occupational health physical examination institutions across 21 cities of Guangdong in South China in 2020. We obtained data on age, sex, enterprise size, work address, and clinical and biochemical indicators of each participant. The dataset includes health examination information of 36,076 lead-exposed workers. We excluded participants carrying out re-examinations (n = 11,517) or pre-job examinations (n = 418) from this study. In addition, participants with missing data on work address (n = 126), blood lead concentration (n = 1879), or blood pressure (n = 134) were also excluded. Finally, 22,002 participants were included (Figure 1). The Ethics Committee of Guangdong Province Hospital for Occupational Disease Prevention and Treatment approved this study.

### 2.2. Outcomes

Systolic and diastolic blood pressure (DBP), and the prevalence of hypertension were the primary outcomes of this study. After a quiet rest of at least 5 min, blood pressure was measured twice, with measurements taken 2 min apart, using an electronic sphygmomanometer. The average of the two measurements was used as the final result. SPB ≥ 140 mmHg and/or DBP ≥ 90 mmHg was defined as hypertension according to 2018 Chinese Guidelines for the Management of Hypertension [19].

### 2.3. Measurement of Blood Lead

Blood lead is a commonly used biomarker that reflects an individual’s recent exposure to lead. All participants had venous blood samples collected after an overnight fasting. Technicians of the physical examination institutions used graphite furnace atomic absorption spectrometry to measure the BLLs. The detection limit was 20 μg/L. The World Health Organization suggested that effective measures should be taken to reduce or terminate lead exposure when BLLs exceed 50 μg/L [20]. Therefore, we chose 20 μg/L and 50 μg/L as cutoff points to divide the blood lead concentration of the participants into 3 levels to investigate the effect of different BLLs on workers’ blood pressure.

### 2.4. Covariates

The covariates involved in this study include basic characteristics of the participants, occupational exposures, air pollutant concentrations, and meteorological factors. Basic characteristics include age, gender, and enterprise size of the company where the participants were employed. Ages were classified into three groups: ≤30, 30–45, and >45 years old, respectively. According to “Statistically classified methods for large, medium, small and micro enterprises (2017)”, the size of enterprises was categorized as micro, small, medium, large, and unknown [21].

Considering that the lead-exposed workers in Guangdong are likely to be simultaneously exposed to productive dust, noise, high temperature, and BTEX, and the effects of these occupational hazards on blood pressure have been reported in previous studies [3,4,5,6], we collected data for these four additional occupational exposures. These variables were defined according to the Classification of Occupational Hazards in the Workplace of Occupational Diseases (GBZ/T229) [22] issued by the Ministry of Health of the People’s Republic of China, and was reported by the enterprise to the key occupational disease monitoring platform of Guangdong Province. Dust exposure was defined as the presence of productive dust in the workplace. Noise exposure referred to a noise in excess of 80 decibels (dB) during an 8 h workday. High temperature exposure meant that the average wet-bulb globe temperature (WBGT) index reaching or exceeding 25 °C in the workplace during production. BTEX exposure referred to exposure to benzene, toluene, ethylbenzene, and xylenes in the workplace.

### 2.5. Air Pollution and Meteorological Exposure

We obtained air pollution data from the ChinaHighAirPollutants (CHAP) dataset. Taking into account the spatiotemporal heterogeneity of air pollution, the dataset is generated from big data such as ground measurements, and model simulations using artificial intelligence. We obtained PM_2.5_, PM_10_, O_3_, SO_2_, and NO_2_ concentrations from this dataset at a spatial resolution of 1 km × 1 km. The cross-validation coefficients (CV-R^2^) for these pollutants were 0.92, 0.90, 0.87, 0.84, and 0.84, respectively. The root-mean-square-errors (RMSE) were 10.76 μg/m^3^, 21.12 μg/m^3^, 17.10 μg/m^3^, 10.07 μg/m^3^, and 7.99 μg/m^3^, respectively. Further details have been described in previous studies [23,24,25,26]. Furthermore, meteorological data were sourced from National Earth System Science Data Center, National Science & Technology Infrastructure of China (http://www.geodata.cn). We obtained monthly temperature and humidity data with a spatial resolution of 1 km × 1 km from this dataset.

We employed the nearest distance matching method, using the geocoding of workplace address of each participant, to obtain monthly data on air pollutant concentrations, temperature, and humidity. Subsequently, we calculated the mean concentrations of pollutants, mean temperature, and humidity for the previous year based on the participants’ monthly average concentration during the 12 months prior to the date of their physical examination.

### 2.6. Statistical Analysis

We described continuous variables and categorical variables in terms of means (standard deviations, SD) and frequencies (percentages), respectively. One-way ANOVA and chi-square test were used to compare inter-group differences for continuous variables and categorical variables, respectively.

Taking into account the potential impact of regulatory policies, economic levels, etc. in different cities, mixed-effect models adjusted for city random intercept were used to evaluate the association of blood pressure and the prevalence of hypertension with BLLs. We developed the following models sequentially:

Model 1: the crude model adjusted for city random intercept;

Model 2: additionally adjusted for basic characteristics and meteorological factors including age, sex (male, female), enterprise size, temperature, and humidity, based on Model 1;

Model 3: additionally adjusted for occupational exposure including dust (yes, no), noise (yes, no), high temperature (yes, no), and BTEX (yes, no), based on Model 3;

Model 4: based on Model 3, additionally adjusted for air pollutants include PM_2.5_, PM_10_, O_3_, SO_2_. and NO_2_.

The multicollinearity issue was assessed using the variance inflation factor, and none of the values exceeded 5, indicating that there was no multicollinearity in our model. In addition, we modeled the BLLs as a continuous variable to test the trend of the effect across lead levels.

To evaluate the potential effect modification, we stratified the results by occupational exposure and the concentrations of pollutants based on the fully adjusted model. Specifically, we divided the continuous pollutants into low- and high-concentration groups using the median as a cutoff point. The cutoff values for PM_2.5_, PM_10_, O_3_, SO_2_, and NO_2_ were 24.52 μg/m^3^, 43.88 μg/m^3^, 103.36 μg/m^3^, 7.26 μg/m^3^, and 28.76 μg/m^3^, respectively.

### 2.7. Sensitivity Analyses

To confirm that the estimates were robust, we conducted several sensitivity analyses, which included excluding participants working in enterprises of unknown size and those with less than one year of service.

All statistical tests were 2-sided, and *p* < 0.05 was considered statistically significant. All analyses were performed using R version 4.2.2.

## 3. Results

### 3.1. Baseline Characteristic

As shown in Table 1, this study included 22,002 participants, of whom 13,278 (60.35%) were male and 8724 (39.65%) were female. The average age of participants was 34.83 years. Among these participants, 2805 (12.75%) had hypertension. The groups with BLLs ≤ 20 μg/L, between 20–50 μg/L, and >50 μg/L consisted of 9415 participants (42.79%), 9000 participants (40.91%), and 3587 participants (16.30%), respectively. The participants in the >50 μg/L group tended to be older, male, or employees of small businesses compared to those in the ≤20 μg/L group. We also found a significantly higher percentage of participants exposed to dust, noise, and high temperature in the >50 μg/L group compared to the ≤20 μg/L group.

In addition, the average concentrations of PM_2.5_, PM_10_, O_3_, SO_2_, and NO_2_ were measured at 24.44 μg/m^3^, 43.52 μg/m^3^, 102.78 μg/m^3^, 7.48 μg/m^3^, and 29.16 μg/m^3^, respectively. The correlation coefficient matrix indicated significant correlations (correlation coefficient > 0.75) between PM_2.5_ and PM_10_, as well as O_3_ (Table S1).

### 3.2. Association of Blood Lead Levels with Hypertension

Table 2 shows the association of blood lead with and SBP and DBP, as well as the prevalence of hypertension. The first two sections display the relationship between BLLs and SBP and DBP (i.e., β), while the third section presents the effect of BLLs on the prevalence of hypertension (i.e., OR). We observed a positive association between BLLs and the prevalence of hypertension (Table 2). In the final model, compared to participants with BLLs ≤ 20 μg/L, those with BLLs between 20–50 μg/L and those >50 μg/L had a higher prevalence of hypertension, with the adjusted odds ratio (OR) and 95% confidence interval (95% *CI*) being 1.26 (95% *CI*: 1.15, 1.40) and 1.37 (95% *CI*: 1.19, 1.57), respectively.

Moreover, we also identified a notable association between BLLs and SBP and DBP. Compared to participants with BLLs ≤ 20 μg/L, those with BLLs between 20–50 μg/L and BLLs > 50 μg/L were associated with a 1.24 (95% *CI*: 0.79, 1.70) mmHg and a 1.27 (95% *CI*: 0.60, 1.93) mmHg increase in SBP, and a 0.65 (95% *CI*: 0.32, 0.98) mmHg and a 1.02 (95% *CI*: 0.54, 1.50) mmHg increase in DBP, respectively.

### 3.3. Potential Modifiers on the Relationship of Blood Lead Levels and Hypertension

Table 3 presents the stratified results based on occupational exposures, revealing no significant interaction between BLLs and these exposures. When stratified by air pollutants (Table 4), we found that participants exposed to high PM_10_ concentrations tended to have a 28% higher OR for hypertension among the 20–50 μg/L group and a 21% higher OR for hypertension among the >50 μg/L group, relative to those exposed to low PM_10_ levels (*P*_interaction_ = 0.04). Meanwhile, we observed significant modification effects of NO_2_ on the relationship between BLLs and the prevalence of hypertension (*P*_interaction_ = 0.04). However, these effects were inconsistent across varying BLLs. A similar trend was also observed in SBP.

In addition, the analysis stratified by the occupational exposures on the association between BLLs and blood pressure showed that workers exposed to high temperatures were more susceptible (Table S2). Compared to workers not exposed to high temperatures, who experienced decreases in SBP of 0.41 mmHg and 2.46 mmHg due to variations in BLLs, those exposed to high temperatures experienced increases in SBP of 1.29 mmHg and 1.65 mmHg, respectively (*P*_interaction_ = 0.02). This trend was also observed in DBP. Workers not exposed to dust exhibited more sensitivity to changes in both SBP and DBP due to variations in BLLs, with *P*_interaction_ values of 0.01 for both SBP and DBP, respectively. Furthermore, workers exposed to high NO_2_ concentrations were more sensitive to changes in DBP due to variations in BLLs (*P*_interaction_ = 0.02) (Table S3).

### 3.4. Sensitivity Analyses

Similar effect estimates were observed in the dataset excluding participants working in enterprises of unknown size (Table S4). Similarly, similar results were shown in a dataset excluding participants with less than one year of service (Table S5).

## 4. Discussion

We found that BLLs were significantly positively associated with blood pressure and the prevalence of hypertension in this study. Currently, the relationship between BLLs and hypertension remains a controversial issue, and the following studies are in agreement with our findings. A cross-sectional study in Haiti found that the group with BLLs exceeding 6.5 μg/dL was associated with a 2.42 mmHg (95% *CI*: 0.36, 4.49) and a 1.96 mmHg (95% *CI*: 0.56, 3.37) increase in SBP and DBP, respectively, relative to the group with below 3.4 μg/dL [27]. A population-based cross-sectional study in Sweden observed that participants with BLLs between 33 μg/L and 258 μg/L demonstrated an increase of 1–2 mmHg in SBP and DBP, and a 30% higher prevalence of hypertension (95% *CI*: 1.1, 1.5), relative to those with BLLs between 1.5–19 μg/L [28]. Several studies in adults over 40 years of age, menopausal women [29], the general population [30], and children [31] also support our findings. However, some previous studies have shown little or no association of BLLs with SBP and DBP and hypertension [11,32]. The variation in the results could potentially be attributed to the differences in the basic characteristics and lifestyle of the different study populations, such as age, diet, and exercise habits. Our study provides further substantiation that supports the association between BLLs and the prevalence of hypertension, suggesting that more effective protective measures are necessary to reduce lead exposure among lead-exposed workers.

Lead exposure may lead to hypertension through multiple pathways. One possible mechanism is that lead may induce oxidative stress by altering the activity of antioxidant enzymes, as well as enhance the generation of reactive oxygen species (ROS), reduce NO bioavailability, and elevate vascular resistance, thereby increasing blood pressure [33,34]. Oxidative stress can also activate transcription factors, promote cellular inflammation and apoptosis, and lead to the occurrence of hypertension [35]. In addition, another potential mechanism of hypertension may be the increased sympathetic tone and increased renin-angiotensin system (RAS) [36,37]. Lead-related hypertension may also be associated with genes such as the M allele in the AGT gene and G allele carriers in the EDN1 gene [38,39]. Lead levels in peripheral blood were also correlated with peripheral artery disease, which may also induce endothelial dysfunction through increased oxidative stress, leading to changes in blood pressure [40]. However, the mechanism needs to be further studied in more detail.

We also observed that participants exposed to higher concentrations of PM_10_ were subjected to stronger blood lead effects on hypertension. One possible reason is that certain components of air pollutants act synergistically with lead to cause hypertension. A study has shown that the combined exposure of lead and black carbon in PM significantly enhances oxidative stress, DNA damage, and the inflammatory response through different interactions and synergistic effects, which may further lead to the occurrence of hypertension [41]. Another possible reason is that air pollutants may react with lead, resulting in the easier absorption of lead into the body and increased lead toxicity [42,43]. However, due to the complexity of the interaction between air pollution and lead exposure, further research is needed to explore the underlying biological mechanisms. We observed the inconsistent modification effects of NO_2_ on the relationship between blood lead and hypertension at different BLLs. Therefore, more data are needed for further investigation.

We observed that participants without dust exposure were more susceptible to blood lead. Participants who were exposed to dust may be less sensitive to occupational harmful factors such as lead than those who are only exposed to lead due to their long-term exposure to more complex working environments. Participants who were occupationally exposed to high temperatures were more likely to be affected by blood lead to SBP. This may be due to the fact that higher temperatures increase lead bioavailability and mobility, leading to increased lead exposure and subsequent lead toxicity [44].

Our study has the following advantages. First, this study included 22,002 participants from 21 prefecture-level cities in Guangdong, South China. The sample size is large enough to be representative of lead-exposed workers and to provide enough statistical power. In addition, we evaluated the interaction effect of BLLs with occupational exposures and air pollutants. To our knowledge, these issues have not been addressed in previous studies. Our findings may provide new ideas for the prevention of hypertension in lead-exposed workers. Last but not least, our study indicated that no level of lead exposure, even below the WHO’s maximum acceptable level of <50 μg/L, can be considered completely free of the potential harm to blood pressure.

Despite these strengths, it is imperative that we acknowledge several limitations. Firstly, as this research is a cross-sectional study, there is an inherent limitation in identifying a clear causal relationship between BLLs and blood pressure. Second, we did not collect information on some covariates such as family history, antihypertensive medication history, lifestyle factors, and dietary factors, which may affect blood pressure. Future work should confirm these findings with more specific data. Third, we did not collect the indoor air pollution data in the workplace. Data on ambient air pollution were matched according to work addresses, potentially introducing misclassification bias and, consequently, underestimating the modification effect of air pollution on the effect of blood lead on hypertension. Finally, in this study, no specific exposure doses for dust, noise, high temperature, and other factors were collected, and more specific data should be used in future studies to confirm these findings.

## 5. Conclusions

In conclusion, our findings suggest the potential negative effect of blood lead on blood pressure and the prevalence of hypertension among workers, with participants exposed to high concentrations of PM_10_, those exposed to occupational high temperature, and those without dust exposure being more vulnerable. These findings suggest that companies and workers should take effective measures to reduce lead exposure to prevent hypertension in workers, taking into account the significance of reducing air pollution and occupational exposure.

## Figures and Tables

**Table 1:** Description of the study participants by blood lead levels.

Characteristics	Overall (N = 22,002)	Blood Lead Levels			
		≤20 μg/L (n = 9415)	20~50 μg/L (n = 9000)	>50 μg/L (n = 3587)	*p*-Value
**Hypertension,** n (%)					<0.001
Yes	2805 (12.75)	1029 (10.93)	1163 (12.92)	613 (17.09)	
No	19,197 (87.25)	8386 (89.07)	7837 (87.08)	2974 (82.91)	
**SBP, mmHg,** mean (SD)	122.85 (14.93)	121.68 (14.57)	123.38 (14.60)	124.63 (16.37)	<0.001
**DBP, mmHg,** mean (SD)	78.11 (10.67)	77.49 (10.47)	78.07 (10.53)	79.82 (11.34)	<0.001
**Age, year,** mean (SD)	34.83 (8.38)	33.41 (7.95)	34.66 (8.09)	38.97 (8.83)	<0.001
**Sex,** n (%)					<0.001
Male	13,278 (60.35)	5306 (56.36)	5519 (61.32)	2453 (68.39)	
Female	8724 (39.65)	4109 (43.64)	3481 (38.68)	1134 (31.61)	
**Enterprise size,** n (%)					<0.001
Micro	464 (2.11)	249 (2.64)	180 (2.00)	35 (0.98)	
Small	6952 (31.60)	2416 (25.66)	2730 (30.33)	1806 (50.35)	
Medium	5942 (27.01)	2880 (30.59)	2584 (28.71)	478 (13.33)	
Large	7434 (33.79)	3818 (40.55)	2604 (28.93)	1012 (28.21)	
Unknown	1210 (5.50)	52 (0.55)	902 (10.02)	256 (7.14)	
**Dust exposure,** n (%)					<0.001
Yes	6987 (31.76)	1625 (17.26)	4136 (45.96)	1226 (34.18)	
No	15,015 (68.24)	7790 (82.74)	4864 (54.04)	2361 (65.82)	
**Noise exposure,** n (%)					<0.001
Yes	2671 (12.14)	968 (10.28)	1104 (12.27)	599 (16.70)	
No	19,331 (87.86)	8447 (89.72)	7896 (87.73)	2988 (83.30)	
**High temperature exposure,** n (%)					<0.001
Yes	1279 (5.81)	346 (3.67)	544 (6.04)	389 (10.84)	
No	20,723 (94.19)	9069 (96.33)	8456 (93.96)	3198 (89.16)	
**BTEX exposure,** n (%)					<0.001
Yes	1030 (4.68)	596 (6.33)	380 (4.22)	54 (1.51)	
No	20,972 (95.32)	8819 (93.67)	8620 (95.78)	3533 (98.49)	
**PM_2.5_, μg/m^3^,** mean (SD)	24.44 (2.33)	24.27 (2.51)	24.50 (2.16)	24.77 (2.23)	<0.001
**PM_10_, μg/m^3^,** mean (SD)	43.52 (3.70)	43.38 (3.93)	43.79 (3.41)	43.19 (3.73)	<0.001
**O_3_, μg/m^3^,** mean (SD)	102.78 (8.15)	103.41 (8.08)	102.35 (8.49)	102.20 (7.27)	<0.001
**SO_2_, μg/m^3^,** mean (SD)	7.48 (1.40)	7.61 (1.14)	7.11 (1.29)	8.07 (1.93)	<0.001
**NO_2_, μg/m^3^,** mean (SD)	29.16 (6.29)	29.06 (6.53)	29.60 (5.70)	28.34 (6.91)	<0.001
**Temperature, °C,** mean (SD)	23.74 (0.43)	23.82 (0.24)	23.80 (0.32)	23.40 (0.75)	<0.001
**Humidity, %,** mean (SD)	73.81 (2.81)	73.96 (2.91)	73.52 (2.80)	74.15 (2.45)	<0.001

Note: Values are mean (SD) or n (%). Abbreviations: SD: standard deviations; SBP: systolic blood pressure; DBP: diastolic blood pressure; PM_2.5_: particulate matter with an aerodynamic diameter ≤ 2.5 μm, PM_10_: particulate matter with an aerodynamic diameter ≤ 10 μm, O_3_: ozone, SO_2_: sulfur dioxide, NO_2_: nitrogen dioxide

**Table 2:** Association of blood lead levels with blood pressure and hypertension.

Outcomes	Model 1	Model 2	Model 3	Model 4
**SBP, *β* (95% *CI*)**				
≤20	Ref. (0)	Ref. (0)	Ref. (0)	Ref. (0)
20–50	2.45 (2.01, 2.88)	1.46 (1.02, 1.89)	1.27 (0.82, 1.71)	1.24 (0.79, 1.70)
>50	4.21 (3.56, 4.86)	1.18 (0.53, 1.83)	1.12 (0.47, 1.78)	1.27 (0.60, 1.93)
***P*_trend_**	*p* < 0.001	*p* < 0.001	*p* < 0.001	*p* < 0.001
**DBP, *β* (95% *CI*)**				
≤20	Ref. (0)	Ref. (0)	Ref. (0)	Ref. (0)
20–50	1.21 (0.90, 1.52)	0.67 (0.35, 0.98)	0.47 (0.15, 0.79)	0.65 (0.32, 0.98)
>50	2.84 (2.37, 3.30)	0.96 (0.49, 1.43)	0.88 (0.40, 1.35)	1.02 (0.54, 1.50)
***P*_trend_**	*p* < 0.001	*p* < 0.001	*p* < 0.001	*p* < 0.001
**Hypertension, *OR* (95% *CI*)**				
≤20	Ref. (1)	Ref. (1)	Ref. (1)	Ref. (1)
20–50	1.36 (1.24, 1.49)	1.21 (1.10, 1.33)	1.22 (1.10, 1.34)	1.26 (1.15, 1.40)
>50	1.98 (1.75, 2.25)	1.32 (1.16, 1.51)	1.33 (1.17, 1.53)	1.37 (1.19, 1.57)
***P*_trend_**	*p* < 0.001	*p* < 0.001	*p* < 0.001	*p* < 0.001

Note: Model 1: the crude model adjusted for city random intercept; Model 2: additionally adjusted for basic characteristics and meteorological factors including age, sex, enterprise size, temperature, and humidity, based on Model 1; Model 3: additionally adjusted for occupational exposure including dust, noise, high temperature, and BTEX, based on Model 3; Model 4: additionally adjusted for air pollutants include PM_2.5_, PM_10_, O_3_, SO_2_, and NO_2_, based on Model 3. Abbreviations: SBP: systolic blood pressure; DBP: diastolic blood pressure.

**Table 3:** Association of blood lead and hypertension, stratified by occupational exposures.

Outcome	Hypertension			
	*OR* (95% *CI*)			*P*_interaction_
Blood Lead Levels, μg/L	≤20	20–50	>50	
**Dust exposure**				
No	Ref. (1)	1.27 (1.02, 1.59)	1.39 (1.07, 1.81)	0.70
Yes	Ref. (1)	1.23 (1.10, 1.38)	1.35 (1.13, 1.60)	
**Noise exposure**				
No	Ref. (1)	1.14 (0.82, 1.57)	1.01 (0.69, 1.49)	0.77
Yes	Ref. (1)	1.27 (1.15, 1.41)	1.41 (1.22, 1.64)	
**High temperature exposure**				
No	Ref. (1)	1.18 (0.70, 1.99)	0.99 (0.57, 1.72)	0.71
Yes	Ref. (1)	1.26 (1.14, 1.39)	1.38 (1.20, 1.59)	
**BTEX exposure**				
No	Ref. (1)	1.04 (0.63, 1.72)	2.57 (0.93, 7.02)	0.31
Yes	Ref. (1)	1.27 (1.15, 1.40)	1.34 (1.17, 1.53)	

Note: The factor used for stratification was excluded from the final model (i.e., Model 4). Abbreviations: OR: odds ratio, CI: confidence interval.

**Table 4:** Association of blood lead and hypertension, stratified by air pollutants concentrations.

Outcome	Hypertension			
	*OR* (95% *CI*)			*P*_interaction_
Blood Lead Levels, μg/L	≤20	20–50	>50	
**PM_2.5_**				
Low	Ref. (1)	1.13 (0.98, 1.31)	1.27 (1.04, 1.55)	0.22
High	Ref. (1)	1.41 (1.23, 1.63)	1.48 (1.22, 1.80)	
**PM_10_**				
Low	Ref. (1)	1.13 (0.97, 1.31)	1.29 (1.07, 1.55)	0.04
High	Ref. (1)	1.41 (1.23, 1.62)	1.39 (1.12, 1.72)	
**O_3_**				
Low	Ref. (1)	1.17 (1.01, 1.37)	1.23 (0.99, 1.52)	0.05
High	Ref. (1)	1.34 (1.18, 1.53)	1.47 (1.22, 1.76)	
**SO_2_**				
Low	Ref. (1)	1.14 (0.98, 1.33)	1.31 (1.07, 1.60)	0.28
High	Ref. (1)	1.32 (1.16, 1.51)	1.30 (1.05, 1.60)	
**NO_2_**				
Low	Ref. (1)	1.17 (1.01, 1.35)	1.34 (1.11, 1.64)	0.04
High	Ref. (1)	1.38 (1.20, 1.59)	1.30 (1.06, 1.60)	

Note: The factor used for stratification was excluded from the final model (i.e., Model 4). Abbreviations: OR: odds ratio, CI: confidence interval, PM_2.5_: particulate matter with an aerodynamic diameter ≤ 2.5 μm, PM_10_: particulate matter with an aerodynamic diameter ≤ 10 μm, O_3_: ozone, SO_2_: sulfur dioxide, NO_2_: nitrogen dioxide.

## Data Availability

The data cannot be made publicly available upon publication because they contain sensitive personal information. The data that support the findings of this study are available upon reasonable request from the authors.

## Supplementary Materials

The following supporting information can be downloaded at: https://www.mdpi.com/article/10.3390/toxics12120861/s1, Table S1. Pearson correlation coefficients for air pollutants; Table S2. Association of blood lead and blood pressure, stratified by occupational exposures; Table S3. Association of blood lead and blood pressure, stratified by air pollutants concentrations; Table S4. Association of blood lead levels with blood pressure and Hypertension in databases without indefinite enterprise size; Table S5. Association of blood lead levels with blood pressure and Hypertension in databases without participants less than one year of service.

## References

[1] Liao L.M., Friesen M.C., Xiang Y.-B., Cai H., Koh D.-H., Ji B.-T., Yang G., Li H.-L., Locke S.J., Rothman N. (2016). Occupational Lead Exposure and Associations with Selected Cancers: The Shanghai Men’s and Women’s Health Study Cohorts. Environ. Health Perspect..

[2] Walter K. (2023). What Is Lead Poisoning?. JAMA.

[3] World Health Organization (2021). The Public Health Impact of Chemicals: Knowns and Unknowns—Data Addendum for 2019.

[4] Chen L., Xie J., Ma T., Chen M., Gao D., Li Y., Ma Y., Wen B., Jiang J., Wang X. (2022). Greenness Alleviates the Effects of Ambient Particulate Matter on the Risks of High Blood Pressure in Children and Adolescents. Sci. Total Environ..

[5] World Health Organization (2021). Guideline for the Pharmacological Treatment of Hypertension in Adults.

[6] Hu S.-S. (2023). Report on Cardiovascular Health and Diseases in China 2021: An Updated Summary. J. Geriatr. Cardiol..

[7] Shen Y., Wang X., Wang Z., Zhang L., Chen Z., Zhu M., Chang C., Gao R. (2018). Prevalence, Awareness, Treatment, and Control of Hypertension among Chinese Working Population: Results of a Workplace-Based Study. J. Am. Soc. Hypertens..

[8] He P., Yang C., He D., Zhao S., Xie Y., Wang H., Ma J. (2021). Blood Lead, Systemic Inflammation, and Blood Pressure: Exploring Associations and Mediation Effects in Workers Exposed to Lead. Biol. Trace Elem. Res..

[9] Bulka C.M., Scannell Bryan M., Persky V.W., Daviglus M.L., Durazo-Arvizu R.A., Parvez F., Slavkovich V., Graziano J.H., Islam T., Baron J.A. (2019). Changes in Blood Pressure Associated with Lead, Manganese, and Selenium in a Bangladeshi Cohort. Environ. Pollut..

[10] Were F.H., Moturi M.C., Gottesfeld P., Wafula G.A., Kamau G.N., Shiundu P.M. (2014). Lead Exposure and Blood Pressure among Workers in Diverse Industrial Plants in Kenya. J. Occup. Environ. Hyg..

[11] Yu Y.-L., Yang W.-Y., Thijs L., Melgarejo J.D., Yu C.-G., Wei D.-M., Wei F.-F., Nawrot T.S., Zhang Z.-Y., Staessen J.A. (2020). Two-Year Responses of Office and Ambulatory Blood Pressure to First Occupational Lead Exposure. Hypertension.

[12] Li X., Wang Y., Liu Q., Sun N., Zhang R., Li X., Yuan J. (2019). Association of occupational heat and noise exposure with hypertension. J. Prev. Med..

[13] Wu Q., Han L., Xu M., Zhang H., Ding B., Zhu B. (2019). Effects of Occupational Exposure to Dust on Chest Radiograph, Pulmonary Function, Blood Pressure and Electrocardiogram among Coal Miners in an Eastern Province, China. BMC Public Health.

[14] Attarchi M., Golabadi M., Labbafinejad Y., Mohammadi S. (2013). Combined Effects of Exposure to Occupational Noise and Mixed Organic Solvents on Blood Pressure in Car Manufacturing Company Workers. Am. J. Ind. Med..

[15] Zhang L., Chen S., Chen Z., Yin W., Fu W., He F., Pan Z., Yi G., Tan X. (2022). Relationship between Occupational Noise Exposure and Hypertension: Cross-Sectional Evidence from Real-World. Front. Public Health.

[16] Morawska L., Allen J., Bahnfleth W., Bennett B., Bluyssen P.M., Boerstra A., Buonanno G., Cao J., Dancer S.J., Floto A. (2024). Mandating Indoor Air Quality for Public Buildings. Science.

[17] Murga A., Kuga K., Yoo S.-J., Ito K. (2019). Can the Inhalation Exposure of a Specific Worker in a Cross-Ventilated Factory Be Evaluated by Time- and Spatial-Averaged Contaminant Concentration?. Environ. Pollut..

[18] Choudhary H., Tarlo S.M. (2014). Airway Effects of Traffic-Related Air Pollution on Outdoor Workers. Curr. Opin. Allergy Clin. Immunol..

[19] (2019). China Hypertension Prevention Guidelines Revision Committee 2018 Chinese guidelines for the management of hypertension. Chin. J. Cardiol..

[20] World Health Organization (2021). WHO Guideline for the Clinical Management of Exposure to Lead.

[21] National Bureau of Statistics of China Statistically Classified Methods for Large, Medium, Small and Micro Enterprises (2017). https://www.stats.gov.cn/sj/tjbz/gjtjbz/202302/t20230213_1902763.html.

[22] (2012). Classification of Occupational Disease Hazards in the Workplace.

[23] Wei J., Li Z., Lyapustin A., Sun L., Peng Y., Xue W., Su T., Cribb M. (2021). Reconstructing 1-Km-Resolution High-Quality PM2.5 Data Records from 2000 to 2018 in China: Spatiotemporal Variations and Policy Implications. Remote Sens. Environ..

[24] Wei J., Li Z., Xue W., Sun L., Fan T., Liu L., Su T., Cribb M. (2021). The ChinaHighPM10 Dataset: Generation, Validation, and Spatiotemporal Variations from 2015 to 2019 across China. Environ. Int..

[25] Wei J., Li Z., Li K., Dickerson R.R., Pinker R.T., Wang J., Liu X., Sun L., Xue W., Cribb M. (2022). Full-Coverage Mapping and Spatiotemporal Variations of Ground-Level Ozone (O_3_) Pollution from 2013 to 2020 across China. Remote Sens. Environ..

[26] Wei J., Li Z., Wang J., Li C., Gupta P., Cribb M. (2023). Ground-Level Gaseous Pollutants (NO_2_, SO_2_, and CO) in China: Daily Seamless Mapping and Spatiotemporal Variations. Atmos. Chem. Phys..

[27] Yan L.D., Rouzier V., Pierre J.L., Lee M.H., Muntner P., Parsons P.J., Apollon A., St-Preux S., Malebranche R., Pierre G. (2022). High Lead Exposure Associated with Higher Blood Pressure in Haiti: A Warning Sign for Low-Income Countries. Hypertension.

[28] Gambelunghe A., Sallsten G., Borné Y., Forsgard N., Hedblad B., Nilsson P., Fagerberg B., Engström G., Barregard L. (2016). Low-Level Exposure to Lead, Blood Pressure, and Hypertension in a Population-Based Cohort. Environ. Res..

[29] Nash D., Magder L., Lustberg M., Sherwin R.W., Rubin R.J., Kaufmann R.B., Silbergeld E.K. (2003). Blood Lead, Blood Pressure, and Hypertension in Perimenopausal and Postmenopausal Women. JAMA.

[30] Qu Y., Lv Y., Ji S., Ding L., Zhao F., Zhu Y., Zhang W., Hu X., Lu Y., Li Y. (2022). Effect of Exposures to Mixtures of Lead and Various Metals on Hypertension, Pre-Hypertension, and Blood Pressure: A Cross-Sectional Study from the China National Human Biomonitoring. Environ. Pollut..

[31] Farzan S.F., Howe C.G., Chen Y., Gilbert-Diamond D., Cottingham K.L., Jackson B.P., Weinstein A.R., Karagas M.R. (2018). Prenatal Lead Exposure and Elevated Blood Pressure in Children. Environ. Int..

[32] Huang Z. (2022). Association Between Blood Lead Level with High Blood Pressure in US (NHANES 1999–2018). Front. Public Health.

[33] Vaziri N.D. (2008). Mechanisms of Lead-Induced Hypertension and Cardiovascular Disease. Am. J. Physiol.-Heart Circ. Physiol..

[34] Farmand F., Ehdaie A., Roberts C.K., Sindhu R.K. (2005). Lead-Induced Dysregulation of Superoxide Dismutases, Catalase, Glutathione Peroxidase, and Guanylate Cyclase. Environ. Res..

[35] Vaziri N., Khan M. (2007). Interplay of Reactive Oxygen Species and Nitric Oxide in the Pathogenesis of Experimental Lead-Induced Hypertension. Clin. Exp. Pharmacol. Physiol..

[36] Toscano C.M., Simões M.R., Alonso M.J., Salaices M., Vassallo D.V., Fioresi M. (2017). Sub-Chronic Lead Exposure Produces Β1-Adrenoceptor Downregulation Decreasing Arterial Pressure Reactivity in Rats. Life Sci..

[37] Simões M.R., Ribeiro Júnior R.F., Vescovi M.V.A., De Jesus H.C., Padilha A.S., Stefanon I., Vassallo D.V., Salaices M., Fioresi M. (2011). Acute Lead Exposure Increases Arterial Pressure: Role of the Renin-Angiotensin System. PLoS ONE.

[38] Kim H.-K., Lee H., Kwon J.-T., Kim H.-J. (2015). A Polymorphism in AGT and AGTR1 Gene Is Associated with Lead-Related High Blood Pressure. J. Renin-Angiotensin-Aldosterone Syst..

[39] Lee H., Kim H.-K., Won H., Im J., Kwon J.-T., Kim H.-J. (2016). Genetic Relationship between an Endothelin 1 Gene Polymorphism and Lead-Related High Blood Pressure. Mol. Cell. Toxicol..

[40] Guallar E., Silbergeld E.K., Navas-Acien A., Malhotra S., Astor B.C., Sharrett A.R., Schwartz B.S. (2006). Confounding of the Relation between Homocysteine and Peripheral Arterial Disease by Lead, Cadmium, and Renal Function. Am. J. Epidemiol..

[41] Jiang N., Wen H., Zhou M., Lei T., Shen J., Zhang D., Wang R., Wu H., Jiang S., Li W. (2020). Low-Dose Combined Exposure of Carboxylated Black Carbon and Heavy Metal Lead Induced Potentiation of Oxidative Stress, DNA Damage, Inflammation, and Apoptosis in BEAS-2B Cells. Ecotoxicol. Environ. Saf..

[42] Baltrusaitis J., Chen H., Rubasinghege G., Grassian V.H. (2012). Heterogeneous Atmospheric Chemistry of Lead Oxide Particles with Nitrogen Dioxide Increases Lead Solubility: Environmental and Health Implications. Environ. Sci. Technol..

[43] Edwards R.D., Lam N.L., Zhang L., Johnson M.A., Kleinman M.T. (2009). Nitrogen Dioxide and Ozone as Factors in the Availability of Lead from Lead-Based Paints. Environ. Sci. Technol..

[44] Levin R., Zilli Vieira C.L., Mordarski D.C., Rosenbaum M.H. (2020). Lead Seasonality in Humans, Animals, and the Natural Environment. Environ. Res..

