# Supplementary material for: The Impact of Blood Lead and Its Interaction with Occupational Factors and Air Pollution on Hypertension Prevalence
Source: Toxics. 2024 Nov 27;12(12):861. doi: 10.3390/toxics12120861 (PMC11679143; doi:10.3390/toxics12120861)
Supplement: Supplementary file 1 [file toxics-12-00861-s001.zip › toxics-3312082-supplementary.pdf]

The impact of blood lead and its interaction with occupational factors and air pollution on hypertension prevalence

Table of Content

Table S1. Pearson correlation coefficients for air pollutants.

Table S2. Association of blood lead and blood pressure, stratified by occupational exposures.

Table S3. Association of blood lead and blood pressure, stratified by air pollutants concentrations.

Table S4. Association of blood lead levels with blood pressure and Hypertension in databases without indefinite enterprise size.

Table S5. Association of blood lead levels with blood pressure and Hypertension in databases without participants less than one year of service.

Table S1. Pearson correlation coefficients for air pollutants.

**Pollutant**	**PM_{25}**	**PM_{10}**	**O_3**	**SO_2**	**NO_2**
**PM_{25}**	1				
**PM_{10}**	0.882	1			
**O_3**	0.763	0.683	1		
**SO_2**	0.434	0.224	0.41	1	
**NO_2**	0.612	0.699	0.395	0.095	1

Abbreviations: $PM_{2.5}$: particulate matter with an aerodynamic diameter of less than 2.5μm, PM_{10}: particulate matter with an aerodynamic diameter of less than 10μm, O_3: ozone, SO_2: sulfur dioxide, NO_2: nitrogen dioxide

Table S2. Association of blood lead and blood pressure, stratified by occupational exposures.

Outcomes	SBP β (95% *CI*)			$P_{interaction}$	DBP β (95% *CI*)			$P_{interaction}$
**Blood Lead Levels, μg/L**	**≤20**	**20-50**	**>50**		**≤20**	**20-50**	**>50**	
**Dust exposure**								
**Yes**	Ref. (0)	0.88(0.34, 1.41)	0.55(-0.32, 1.42)	0.01	Ref. (0)	0.30(-0.08, 0.69)	0.68(0.05, 1.30)	0.01
**No**	Ref. (0)	1.72(0.74, 2.70)	2.53(1.34, 3.71)		Ref. (0)	1.16(0.46, 1.86)	1.79(0.94, 2.64)	
**Noise exposure**								
**Yes**	Ref. (0)	1.23(0.75, 1.72)	1.55(0.81, 2.28)	0.64	Ref. (0)	0.69(0.34, 1.03)	1.33(0.81, 1.86)	0.16
**No**	Ref. (0)	1.18(-0.15, 2.51)	0.21(-1.43, 1.86)		Ref. (0)	0.38(-0.58, 1.33)	-0.30(-1.48, 0.88)	
**High temperature exposure**								
**Yes**	Ref. (0)	1.29(0.82, 1.76)	1.65(0.95, 2.35)	0.02	Ref. (0)	0.62(0.29, 0.96)	1.17(0.66, 1.67)	0.16
**NO**	Ref. (0)	-0.41(-2.56, 1.75)	-2.46(-4.87, -0.05)		Ref. (0)	0.25(-1.30, 1.81)	-0.75(-2.50, 1.01)	
**BTEX exposure**								
**Yes**	Ref. (0)	1.21(0.75, 1.68)	1.20(0.53, 1.88)	0.98	Ref. (0)	0.67(0.34, 1.01)	0.94(0.46, 1.43)	0.15
**NO**	Ref. (0)	1.25(-0.78, 3.29)	3.00(-1.70, 7.70)		Ref. (0)	-0.09(-1.58, 1.41)	3.18(-0.26, 6.63)	

Note: The factor used for stratification was excluded from the final model (i.e., model 4).

Abbreviations: SBP: systolic blood pressure; DBP: diastolic blood pressure; $PM_{2.5}$: particulate matter with an aerodynamic diameter of less than 2.5μm, PM_{10}: particulate matter with an aerodynamic diameter of less than 10μm, O_3: ozone, SO_2: sulfur dioxide, NO_2: nitrogen dioxide

Table S3. Association of blood lead and blood pressure, stratified by air pollutants concentrations.

Outcome	SBP				DBP			
	β (95% *CI*)			$P_{interaction}$	β (95% *CI*)			$P_{interaction}$
**Blood Lead Levels, μg/L**	**≤20**	**20-50**	**>50**		**≤20**	**20-50**	**>50**	
**$PM_{2.5}$**								
**Low**	Ref. (0)	1.06(0.41, 1.72)	1.78(0.80, 2.75)	0.11	Ref. (0)	0.38(-0.09, 0.84)	1.00(0.30, 1.69)	0.06
**High**	Ref. (0)	1.31(0.66, 1.95)	0.64(-0.30, 1.58)		Ref. (0)	0.86(0.39, 1.32)	0.80(0.12, 1.48)	
**PM_{10}**								
**Low**	Ref. (0)	0.74(0.06, 1.43)	1.29(0.35, 2.23)	0.02	Ref. (0)	0.30(-0.19, 0.78)	0.75(0.09, 1.41)	0.01
**High**	Ref. (0)	1.50(0.88, 2.12)	0.71(-0.28, 1.71)		Ref. (0)	1.01(0.56, 1.46)	1.02(0.30, 1.75)	
**O_3**								
**Low**	Ref. (0)	1.31(0.62, 1.99)	1.52(0.52, 2.51)	0.19	Ref. (0)	0.51(0.03, 1.00)	0.77(0.06, 1.47)	0.85
**High**	Ref. (0)	1.18(0.56, 1.79)	1.01(0.09, 1.93)		Ref. (0)	0.79(0.34, 1.24)	1.11(0.45, 1.78)	
**SO_2**								
**Low**	Ref. (0)	0.94(0.28, 1.59)	2.13(1.23, 3.04)	0.10	Ref. (0)	0.50(0.03, 0.97)	1.47(0.82, 2.12)	0.08
**High**	Ref. (0)	1.41(0.75, 2.08)	-0.39(-1.47, 0.69)		Ref. (0)	0.54(0.06, 1.01)	-0.07(-0.84, 0.70)	
**NO_2**								
**Low**	Ref. (0)	0.87(0.19, 1.56)	1.28(0.28, 2.28)	0.02	Ref. (0)	0.15(-0.34, 0.63)	0.69(-0.02, 1.40)	0.02
**High**	Ref. (0)	1.42(0.80, 2.04)	0.78(-0.16, 1.72)		Ref. (0)	1.09(0.65, 1.54)	0.93(0.25, 1.61)	

Note: The factor used for stratification was excluded from the final model (i.e., model 4).

Abbreviations: SBP: systolic blood pressure; DBP: diastolic blood pressure; $PM_{2.5}$: particulate matter with an aerodynamic diameter of less than 2.5μm, PM_{10}: particulate matter with an aerodynamic diameter of less than 10μm, O_3: ozone, SO_2: sulfur dioxide, NO_2: nitrogen dioxide

Table S4. Association of blood lead levels with blood pressure and Hypertension in databases without indefinite enterprise size.

Blood Lead Levels, μg/L	SBP *β* (95% *CI*)	DBP *β* (95% *CI*)	Hypertension *OR* (95% *CI*)
**≤20**	Ref. (0)	Ref. (0)	Ref. (1)
**20-50**	1.32(0.86, 1.78)	0.68(0.36, 1.01)	1.28(1.16, 1.41)
**>50**	1.04(0.35, 1.73)	0.93(0.44, 1.43)	1.36(1.18, 1.57)
**P for trend**	*P*<0.001	*P*<0.001	*P*<0.001

Note: model adjusted for age, sex, enterprise size, temperature and humidity, dust exposure, noise exposure, high temperature exposure, and BTEX exposure, $PM_{2.5}$, PM_{10}, O_3, SO_2 and NO_2.

Table S5. Association of blood lead levels with blood pressure and Hypertension in databases without participants less than one year of service.

Blood Lead Levels, μg/L	SBP β (95% *CI*)	DBP β (95% *CI*)	Hypertension *OR* (95% *CI*)
**≤20**	Ref. (0)	Ref. (0)	Ref. (1)
**20-50**	1.22(0.67, 1.77)	0.82(0.42, 1.22)	1.30(1.16, 1.46)
**>50**	1.33(0.44, 2.23)	1.02(0.37, 1.67)	1.22(1.01, 1.47)
**P for trend**	P<0.001	P<0.001	P<0.001

Note: model adjusted for age, sex, enterprise size, temperature and humidity, dust exposure, noise exposure, high temperature exposure, and BTEX exposure, $PM_{2.5}$, PM_{10}, O_3, SO_2 and NO_2.
